# Developmental Trajectories of Nonsuicidal Self-Injury and Risk for Suicide Attempt

**DOI:** 10.1016/j.jaacop.2025.10.004

**Published:** 2025-10-15

**Authors:** Amanda J. Thompson, Katherine Sarkisian, Elyse N. Llamocca, Christopher C. Henrich, Jennifer L. Hughes, Eric A. Youngstrom, Donna A. Ruch, Jeffrey A. Bridge, Cynthia A. Fontanella

**Affiliations:** a The Center for Suicide Prevention and Research, Abigail Wexner Research Institute at Nationwide Children’s Hospital, Columbus, Ohio; b The Ohio State University College of Medicine, Columbus, Ohio; c University of Alabama at Birmingham, Birmingham, Alabama; d Center for Health Policy and Health Services Research, Henry Ford Health, Detroit, Michigan

**Keywords:** developmental trajectories, nonsuicidal self-injury, NSSI, self-injury, suicide attempt

## Abstract

**Objective:**

Suicide attempt (SA) risk is especially high among youth with early nonsuicidal self-injury (NSSI) onset and persistent NSSI. Still, few youth experience persistent NSSI, and few attempt suicide. Identifying which youth follow specific NSSI trajectories and which NSSI trajectories are at higher risk for SA has strong potential to inform more targeted early suicide risk identification and prevention. The present study aimed to identify NSSI trajectories, identify characteristics forecasting which NSSI trajectories youth followed, and compare SA risk across trajectories.

**Method:**

A subsample of youth (N = 2,524) with at least 1 NSSI event before typical onset was retrospectively identified. Youth were followed for 4 years (ages 9-14 years) using the first 5 annual assessments from the Adolescent Brain Cognitive Development℠ (ABCD) Study (release 5.1).

**Results:**

Latent-class growth modeling identified 2 subgroups of youth following distinct NSSI trajectories. The earlier-onset group (15%, mean [SD] age at onset = 9.83 [0.59] years) experienced baseline limited NSSI. The later-onset group (85%, mean [SD] age at onset =11.63 [1.60] years) had moderate risk for more than 1 NSSI endorsement. The later-onset group was significantly more likely to attempt suicide than the earlier-onset group (21% vs. 17% reported ≥1 SAs). Sex, psychopathology, family conflict, and positive parenting predicted group membership and SA risk.

**Conclusion:**

SA risk among youth with early-onset or persistent NSSI was high; however, risk was slightly higher for youth with persistent NSSI. Whereas youth and family characteristics may forecast which NSSI trajectories youth follow, clinical implications of this research support children with NSSI are at risk for SA and may need continued monitoring and intervention. Findings support promoting broad public health awareness of SA risk in youth with NSSI.

## Plain language summary

Using longitudinal data from the Adolescent Brain Cognitive Development℠ (ABCD) study, this study aimed to identify factors associated with nonsuicidal self-injury (NSSI) and suicide attempt among youth with at least one NSSI event prior to age 14 (N = 2,524). Youth with later onset of NSSI (mean age 11.63 years) were more likely to report multiple episodes of NSSI and to attempt suicide than youth with earlier-onset NSSI (mean age 9.83 years). Sex, mental health problems, family conflict, and positive parenting were associated with age of onset of NSSI and risk of suicide attempt.

Nonsuicidal self-injury (NSSI), or purposefully harming one’s body without suicidal intentions, is among the strongest predictors of suicide attempt (SA) and suicide death.^1^, ^2^, ^3^ Among community samples, about 9% of children (age ≤11 years) and 20% of adolescents have a lifetime history of NSSI.^4^^,^^5^ The association between NSSI and SA is especially potent during adolescence.^1^^,^^3^ Still, SA occurs much less often than NSSI, with 3% of children^5^ and 10% of adolescents^6^ reporting a lifetime history of SA. Children (generally referred to as age <12 years) and adolescents with early-onset NSSI (age <14 years) are at especially high risk for SA as well as for increasingly medically severe NSSI that extends into adolescence.^1^^,^^7^ Despite youth with early-onset NSSI having high risk for SA, no study has examined NSSI trajectories beginning in childhood through early adolescence or considered whether children who follow certain NSSI trajectories are at greater risk for SA. Thus, current understanding of which youth who experience NSSI are also most likely to experience SA is extremely limited. To address this research gap and effectively inform interventions aimed at shifting children away from NSSI and SA, it is critical to identify developmental NSSI trajectories spanning before and after typical ages of NSSI onset, identify early-emerging predictors of NSSI trajectories, and compare SA risk across NSSI trajectories.

### Trajectories of NSSI

In the present study, we define self-injury as encompassing suicidal self-injury and NSSI; we refer to NSSI and SA as distinct constructs that often co-occur. NSSI is a global issue, and the average age for NSSI onset (approximately 14 years) may be trending younger.^8^^,^^9^ NSSI affects about 20% and 40% of adolescents from community and clinical samples, respectively.^10^ Several studies have identified different trajectories of NSSI across adolescence, which helps to provide important context about how NSSI typically unfolds across adolescence and whether certain patterns are more or less common for youth with certain traits and characteristics. Similar NSSI trajectories have emerged across studies suggesting that most adolescents do not experience NSSI or experience NSSI that resolves without intervention, but a smaller proportion of adolescents engage in more frequent NSSI or medically severe NSSI.^11^, ^12^, ^13^, ^14^ Adolescents who experience NSSI before 12 years or NSSI persisting up to and beyond 1 year are at high risk for medically severe NSSI, SA, and having more than 1 SA.^1^^,^^7^^,^^15^ Although significantly less is known about children, 1 prospective study found that among children with depression, about half experiencing self-injurious thoughts or behaviors (SITB)—which span thoughts about self-harm and self-harming behavior regardless of suicidal intention—during adolescence first experienced SITB in early childhood.^16^ Notably, adolescents with earlier NSSI onset tend to engage in NSSI more often and with more varied methods,^17^ and they more often engage in suicidal planning.^1^ However, the continuous unfolding of NSSI as children transition into adolescence is poorly understood in terms of persistence of risk or change in behavior. Although earlier NSSI onset portends higher risk for more medically severe self-injury, our present understanding of NSSI trajectories that begin before typical onset is extremely limited. Therefore, an important first step to understanding how NSSI unfolds across development and who is most at risk for NSSI that persists or co-occurs with SA is to examine these patterns beginning in childhood. To date, most studies have focused on NSSI during adolescence (eg, age ≥12 years) or SITB broadly.^18^^,^^19^

### Predictors of NSSI Trajectories

NSSI is theorized to have multiple functions including intrapersonal (eg, regulating negative emotions) or interpersonal (eg, escaping uncomfortable social situations) functions.^20^ Persistent NSSI tends to be associated with intrapersonal functions; thus youth with emotion dysregulation with NSSI that functions to regulate affect may especially be at greater risk for persistent NSSI.^8^^,^^20^^,^^21^ Risk factors for NSSI and SA often overlap among adolescents, including emotion dysregulation, depression, impulsivity, family conflict,^8^^,^^22^^,^^23^ and coercive parenting behaviors.^24^ Although less is known about childhood NSSI, both children and adolescents share similar risk factors, including impulsivity, irritability, depression, exposure to violence, emotion dysregulation, family conflict, and maltreatment.^19^^,^^25^^,^^26^ Some risk factors such as impulsivity and temperament traits are largely heritable, show some stability, and can often be measured at younger ages. Traits such as negative urgency—tendencies to act impulsively when experiencing strong negative affect—have been associated with risk for more severe and persistent NSSI and may be more sensitive to detecting NSSI risk than broad impulsivity.^23^^,^^27^ Behavioral inhibition and activation are neurobiological systems that regulate sensitivity and response to punishment and reward, respectively.^28^^,^^29^ Greater levels of both behavioral inhibition and behavioral activation are associated with both emotion dysregulation and risk for NSSI.^28^^,^^29^ Thus, youth with temperament traits associated with emotion dysregulation in the context of high family conflict and low levels of support from caregivers may be especially at risk for NSSI and SA.

### Risk for SA across NSSI Trajectories

Prior research strongly supports an increased risk for SA and suicide among adolescents with NSSI.^1^, ^2^, ^3^ In a sample of psychiatrically hospitalized adolescents, 70% of those with NSSI also attempted suicide, 55% of whom endorsed multiple SAs.^30^ In one of the few studies examining self-injury in preschool-age children with depression, 19% reported NSSI with suicidal ideation (SI) and/or suicidal behavior, whereas 15% reported NSSI only.^25^ NSSI and SA typically occur in close temporal proximity with variable order of onset. Yet, most studies examine only a unilateral transition of NSSI to SA.^31^, ^32^, ^33^ Some studies have specifically excluded youth with a history of suicidal thoughts or behavior that emerged before NSSI, thus examining only 1 possible pathway—NSSI to SA.^32^ Moreover, 1 study among adults found SA before NSSI rather than the reverse occurred in 9% of the sample.^34^ Thus, unilateral transition from NSSI to SA is unlikely to generalize to everyone. Although NSSI often emerges before SA, many youth with NSSI never transition to suicidal behavior, and some youth experience persistent NSSI and more than 1 SA. NSSI is a strong risk factor for SA, but it is not clear which youth will experience both NSSI with SA. This lack of research on youth with NSSI that co-occurs with SA across development is especially problematic given that youth with early-onset and persistent NSSI are at even higher risk for SA.^7^^,^^31^ Thus, research identifying which youth are at risk of following NSSI trajectories that are characterized by engaging in NSSI more frequently, engaging in NSSI for longer periods of time, or experiencing NSSI with SA has strong potential to inform early-risk identification efforts about which youth are at risk of SA.

### Present Study

Prior research suggests that most adolescents do not engage in NSSI, some engage in NSSI briefly, and very few persistently engage in NSSI.^11^, ^12^, ^13^, ^14^^,^^35^ These subgroups of adolescents follow different patterns of NSSI behavior across early through later adolescence—or different trajectories. It remains unclear why some youth experience earlier onset of NSSI or why some of these youth also attempt suicide. Similar to many prior studies, we leverage a sophisticated modeling technique, latent class growth curve analysis (LCGA), that identifies homogeneous subgroups of youth who follow similar trajectories of NSSI behavior over time.^11^, ^12^, ^13^, ^14^^,^^35^ These models assume that homogeneous subpopulations exist within the overall heterogeneous population; clarifying these subgroups then allows us to identify which trajectories are at higher risk for SA and characteristics of youth following higher-risk trajectories.^36^ This study uniquely follows youth beginning at much younger ages while not excluding youth based on prior history of SITB. We hypothesized that predictors of youth NSSI identified in prior research including greater family conflict, negative urgency, behavioral inhibition, and lower behavioral activation and caregiver acceptance would forecast which youth follow NSSI trajectories at greater risk for SA.^8^^,^^19^

## Method

### Data

The present study is a secondary data analysis using the baseline and the first 4 annual follow-up assessments of the Adolescent Brain Cognitive Development℠ (ABCD) Study (5.1 release; doi.org/10.15154/z563-zd24), which included 11,868 youth–caregiver dyads. ABCD Study® data were collected across 20 US cities beginning in 2017 when youth were 9 years old. Additional study details are described elsewhere.^37^

### Sample Selection

Given our interest in identifying early emerging predictors of risk for SA across trajectories of youth NSSI, our sample uniquely focuses on risk for SA among youth with early NSSI onset. We retrospectively identified 2,524 youths with at least 1 NSSI endorsement during the study period; endorsement could include lifetime history of NSSI at baseline (93% of the sample endorsed NSSI before age 14 years). Baseline predictors between youth with and youth without NSSI were compared, and patterns of missing data were analyzed (see Supplement 1, available online, for description of these differences and accompanying Table S1, available online).

#### Baseline Covariates

We included caregiver report of their child’s biological sex assigned at birth, race/ethnicity, and age in years rounded to the nearest tenth at baseline as covariates. Because SA and NSSI were rare within certain racial groups at certain assessments, we collapsed racial groups into categories with cell sizes ≥10 in accordance with ABCD data use guidelines. Race was thus defined as non-Hispanic Black, Hispanic, non-Hispanic White, and other (defined as non-Hispanic multiracial, American Indian/Alaska Native, Asian American, or Native Hawaiian and other Pacific Islander).

#### Baseline Predictors of Developmental NSSI Trajectories

We used the following baseline variables to predict which youth were more likely to follow certain trajectories:

1. Youth and/or caregiver-reported SI using affirmation of current (≤2 weeks before assessment) and/or lifetime history (>2 weeks) from the suicidality module of the Schedule for Affective Disorders and Schizophrenia for School-Age Children–Present and Lifetime version (K-SADS-PL)^38^
2. Caregiver report of youth’s broad internalizing problems T scores using the Child Behavior Checklist, which measures emotional and behavioral problems such as depression, withdrawal, and anxiety^39^
3. Caregiver’s mean reported financial hardships using a material deprivation scale^40^
4. Youth’s self-reported mean levels of negative urgency,^23^^,^^41^ behavioral activation,^42^ and behavioral inhibition^42^
5. Parental acceptance (positive social interactions with primary caregiver) using the Parental Behavior Inventory^43^
6. Family conflict using the Family Environment Scale.^44^

#### NSSI Trajectories

Youth completed the 35-item computerized version of the K-SADS-PL at each assessment; caregivers completed the suicidality module at every other assessment. Because participants often do not disclose lifetime self-injury history at follow-up or do not disclose self-injury for fear of safety protocols or stigma, we included both caregiver and child positive endorsements of NSSI.^45^ The suicidality module asks, “Sometimes when kids get upset or feel numb, they may do things to hurt themselves, like scratching, cutting, or burning themselves. In the past 2 weeks, how often have you done any of these things or other things to try to hurt yourself?” Positive endorsement of this item was followed by questions about suicidal intentions: “You mentioned that in the past 2 weeks, you did some things to hurt yourself, like scratching, cutting, or burning yourself. Were you trying to kill yourself when you did these things?” Summary diagnoses of current NSSI (within the past 2 weeks) and past NSSI (at any time prior) are defined using these items; in the present study, we define positive NSSI endorsements as youth and/or parent disclosure of NSSI. A limitation to this K-SADS-PL suicidality module is such that youth and caregivers were asked about current and lifetime history of SITB, not whether SITB occurred since the last assessment. Thus, NSSI lifetime history endorsement during follow-up could have occurred at any time prior (including before baseline). At baseline, 24% of youth in the study sample (*n* = 597) disclosed that NSSI occurred at some time in the past, but these youth did not report that the behavior had occurred within the past 2 weeks. Other youth in the study sample (*n* = 1927, 76%) both disclosed that NSSI occurred at some time in the past and endorsed the item that NSSI had occurred within the past 2 weeks (defined as current). These data suggest some youth had previously experienced NSSI that was not current at baseline. To account for these differences, baseline lifetime NSSI history was treated as a covariate; current baseline NSSI was included as 1 of 5 time points in the trajectory. Youth could have ≤5 positive NSSI endorsements, excluding lifetime NSSI history at baseline. We define NSSI onset as the participant’s age at the assessment when NSSI was first endorsed, and thus the age is an estimate and not exact to the moment of onset.

#### Risk for SA Across NSSI Trajectories

Risk for SA was defined as any parent and/or youth positive endorsement of SA at any assessment (≥1 SA) including lifetime history at baseline. To define the total number of SAs, we followed the same logic used to define the frequency of NSSI. More specific details on how self-injury was defined, including all items defining SA and NSSI from the ABCD Study, are presented in Table S2, available online.

### Data Analysis

#### Missing Data

We used full information maximum likelihood estimation methods with robust standard errors to address missing data using Mplus 8, which assumes data are unlikely to be missing completely at random and produces estimates based on all available data.^46^ See Supplement 3, available online, for missing data analyses.

First, linear and quadratic latent growth models were fit. Intercepts and slopes were examined for significant between-person variances. Next, LCGA was used to identify distinct NSSI trajectories. Mean (SD) time between assessments ranged from 0.91 (0.24) to 1.2 (0.24) years. To account for individually varying time between assessments, factor loadings were fixed at the number of years passed since baseline rounded to the nearest tenth. We used traditional fit indices to determine the number of latent class trajectories including Bayesian information criterion (BIC), Akaike information criterion (AIC), consistent AIC (CAIC), Lo-Mendell-Rubin likelihood ratio test, bootstrapped likelihood ratio test, and entropy.^36^ We then used the manual 3-step Bolck, Croon, and Hagenaars (BCH) method, which assigns youth to their most likely trajectory or latent class, accounts for uncertainty in class membership, and then tests the effects of predictors and covariates on both risk of SA and latent class membership.^47^ An omnibus Wald χ^2^ test was used to test for differences in SA risk across trajectory classes. Tests for post hoc pairwise intercept differences were specified using model constraints. Significant differences between class-specific intercepts indicate differences in SA risk across trajectories.^47^

## Results

### Sample Characteristics

The sample with NSSI (*N* = 2,524) was nearly equal male and female (51.8%), more than half were non-Hispanic White (54%), and youth were about 10 years old at baseline (mean [SD] = 9.92 [0.62]) and 14 years old at the last assessment (mean [SD] = 14.08 [0.68]). Table 1 shows demographics and frequencies of SITB over time. Age at NSSI onset was mean (SD) 11.35 (1.63) years. Most youth (87%) endorsed NSSI once, 11% endorsed NSSI at 2 assessments, and 2% endorsed NSSI at ≥3 assessments. Eleven youths (<1%) reported ≥4 NSSI endorsements including baseline lifetime. At baseline, 6% endorsed SA history. By the end of the study, 20% endorsed ≥1 SAs.

**Table 1:** Sample Characteristics

	N	n	(%)
Demographics			
Sex, female	2,523	1,381	(51.8)
Black	2,522	308	(12.0)
Hispanic	2,522	495	(20.0)
Other	2,522	349	(14.0)
White	2,522	1,370	(54.0)
Self-injurious thoughts and behavior			
Baseline NSSI history	2,519	822	(32.6)
Baseline NSSI current	2,519	473	(18.8)
NSSI 1-y FU	2,491	429	(17.2)
NSSI 2-y FU	2,471	570	(23.6)
NSSI 3-y FU	2,192	473	(21.6)
NSSI 4-y FU	1,138	345	(30.3)
Baseline ideation	2,524	795	(31.5)
Baseline SA	2,517	141	(4.5)
SA 1-y FU	2,472	93	(3.8)
SA 2-y FU	2,417	114	(4.7)
SA 3-y FU	2,209	105	(4.8)
SA 4-y FU	1,135	94	(8.3)

Note: FU = follow-up (since baseline); NSSI = nonsuicidal self-injury; SA = suicide attempt.

### Developmental Trajectories of NSSI

Results from the unconditional LCGA (N = 2,524) indicated that a 2-class quadratic solution fit the data best (Table S3, available online). The BIC and CAIC decreased with the addition of a third and fourth class, suggesting improved model fit. However, entropy decreased with the addition of a third and fourth class, suggesting poor separation or that trajectories were less distinct. The bootstrapped likelihood ratio test also did not support significant improvement with the addition of a fourth class. Further inspection of the 3-class solution showed no substantive differences between 2 of the 3 classes (Table 2). Thus, a 2-class solution was selected as the best-fitting model.

**Table 2:** Latent-Class Trajectory Model Identification and Fit Statistics

No. classes	Entropy	LMR LRT	Boot	AIC	BIC	CAIC
1	—	—	—	11,049.16	11,066.66	11,057.12
2	0.87	0.00	0.00	10,815.56	10,856.40	10,834.16
3	0.62	0.00	0.43	10,694.93	10,759.10	10,724.15
4	0.48	0.00	0.25	10,980.26	11,068.62	11,020.96

Note: AIC = Akaike information criterion; BIC = Bayesian information criterion; CAIC = consistent AIC; LMR LRT = Lo-Mendell-Rubin likelihood ratio test.

**Figure 1:**
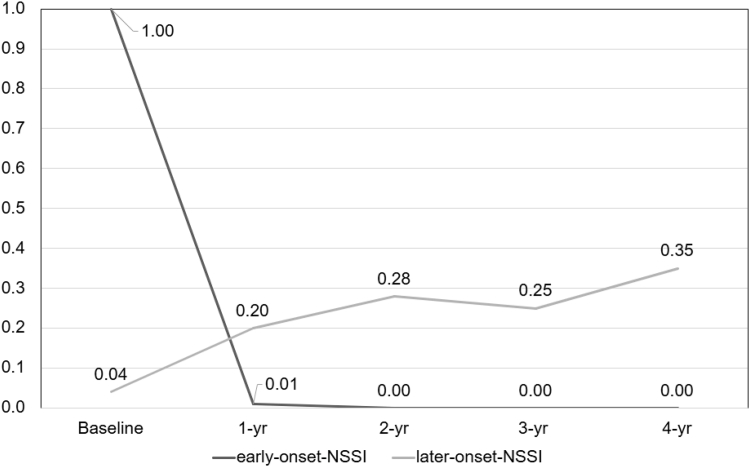
Latent Trajectories of Nonsuicidal Self-Injury in High-Risk Groups

Class 1 (15%, n = 386) was more likely to report earlier onset of NSSI with rapidly declining risk for NSSI over time. Class 2 (85%, n = 2,138) was more likely to report older onset and was characterized by increasing risk for NSSI over time that peaked at the final assessment. See Figure 1 for observed NSSI trajectories. Posterior probability estimates were used to examine the onset and frequency of NSSI endorsements across trajectories. Mean (SD) NSSI onset was earlier in class 1 (9.83 [0.59], *SD*=0.59) than in class 2 (11.63 [1.60]). In class 1, 99% of youth endorsed current NSSI at the first assessment, and 1% endorsed NSSI at 2 assessments. In class 2, 85% of youth endorsed NSSI only once (24% of whom endorsed baseline lifetime history only with no subsequent endorsements), 13% endorsed NSSI at 2 assessments, and 2% endorsed at ≥3 assessments. For ease of interpretation, we named class 1 the earlier-onset group and class 2 the later-onset group.

### Predictors of NSSI Trajectories

The multinomial logistic regression from the conditional BCH model (*n* = 2,506) found that youth assigned to the earlier-onset group compared with the later-onset group were more likely to be younger and male. The earlier-onset group compared with the later-onset group was also more likely to have higher internalizing problems, a baseline lifetime history of NSSI and SI, more family conflict, and lower parental acceptance. Additional results and odds ratios are listed in Table 3.

**Table 3:** Multinomial Logistic Regression: Earlier-Onset Group vs Later-Onset Group

	OR	SE	95% CI
Demographics			
Age	0.69	0.09	(0.55, 0.89)
Black	1.38	0.33	(0.86, 2.19)
Hispanic	1.40	0.28	(0.95, 2.07)
Other	1.06	0.26	(0.67, 1.72)
NSSI history	1.72	0.29	(1.24, 2.39)
Sex, female	0.44	0.08	(0.31, 0.62)
Predictors			
Behavioral inhibition	1.08	0.12	(0.88, 1.35)
Behavioral activation	1.22	0.17	(0.93, 1.60)
Family conflict	1.11	0.04	(1.04, 1.19)
Ideation	1.61	0.28	(1.15, 2.27)
Internalizing	1.03	0.01	(1.01, 1.04)
Material deprivation	1.38	0.57	(0.96, 1.08)
Negative urgency	1.02	0.03	(0.96, 1.08)
Parental acceptance	0.64	0.14	(0.42, 0.98)

Note: All variables were measured at baseline. Non-Hispanic White is the reference group. Sex reference group is male. Table should be interpreted as the odds of youth assigned to class 2 in reference to class 1. NSSI = nonsuicidal self-injury; OR = odds ratio; SE = standard error.

### Risk for SA Across NSSI Trajectories

The omnibus Wald test revealed statistically significant, yet small, differences in risk for SA across the groups (*χ*^2^_1_ = 19.18, p < .001, *ϕ* = 0.09). Pairwise comparisons indicated a significantly lower mean difference in SA risk between the earlier-onset group and the later-onset group (mean difference = −0.12). It should be noted that the regression of SA risk and class membership were included in the BCH model; thus, these differences account for all covariates (eg, sex, race/ethnicity). Using posterior probabilities, we found that 83% of the earlier-onset group reported no SA, 15.5% reported 1 SA, and 1.5% reported ≥2 SAs. In the later-onset group, 79% reported no SA, 20% reported 1 SA, and 1% reported ≥2 SAs. Of the earlier-onset group, 11% (*n* = 44) entered the study at baseline with a history of at least 1 SA, and 6.5% (n = 25) endorsed at least 1 SA during follow-up. Of the later-onset group, 4.5% (*n* = 97) entered the study at baseline with a history of at least 1 SA, and 17% (n = 372) endorsed at least 1 SA during follow-up.

## Discussion

The present study identified 2 distinct NSSI trajectories spanning middle childhood into mid-adolescence. We hypothesized that trajectories would differ in SA risk based on existing literature examining self-injury among adolescent and child populations.^8^^,^^19^ The earlier-onset group (15%) followed a trajectory characterized by high risk for a singular baseline endorsement of NSSI with low risk for subsequent NSSI and lower risk for SA (17% reported ≥1 SAs). The *later-onset group* (85%) followed a trajectory characterized by older NSSI onset, moderate risk for subsequent NSSI endorsements that increased over time, and higher SA risk (21% reported ≥1 SAs). Although NSSI trajectories and characteristics of youth following these trajectories were distinct, differences in SA risk across trajectories were not meaningfully distinct. This small difference in SA risk could be attributable to the fact that nearly all youth (93%) in the present study experienced early-onset NSSI (age <14 years). More broadly, these findings add to the growing concern that youth with early-onset NSSI are at high risk for SA, and certain youth and family characteristics may help identify risk factors for NSSI that emerges during childhood.

The younger NSSI onset in the earlier-onset group and the risk for persistence in the later-onset group could be attributable to sex differences. Peak age of SITB onset tends to be earlier among boys, and the prevalence of SITB tends to be higher among boys during childhood, whereas prevalence is higher among girls during adolescence.^19^^,^^48^ Although mechanisms explaining developmental differences in SITB risk across sex are complex and multifaceted, age- and sex-specific patterns for certain mental health conditions may help to explain some of these differences.^48^ The higher risk for persistence and SA in the later-onset group is supported by existing research suggesting that girls are disproportionately at risk for persistent NSSI and NSSI with SA.^24^^,^^48^ Sex-specific differences in NSSI function are associated with NSSI persistence, and more research is needed on functions of NSSI across sex in youth at younger ages. Girls more commonly report intrapersonal NSSI functions, which are associated with more persistent NSSI and NSSI with SA.^20^^,^^21^^,^^24^ Presently, childhood NSSI functions are understudied. Moreover, sex-specific differences in risk for childhood NSSI separate from other forms of self-injury or SITB broadly are unclear.^19^

Aligned with prior literature, we found that baseline higher family conflict, lower caregiver acceptance, history of SI, and more internalizing problems predicted greater odds that youth belonged to the *earlier-onset group* and may highlight unique risk factors for childhood-onset NSSI.^19^ Family conflict, which includes shouting or throwing things to express anger, may be especially harmful to younger youth who are heavily dependent on caregivers to provide a safe environment and to support developing emotion regulation in children. More adaptive parenting is also associated with lower risk for NSSI.^49^ Thus, higher baseline parental acceptance might explain the delayed onset of NSSI in the later-onset group.

It remains challenging to identify and intervene with youth at risk of self-injury. Caregivers and clinicians may be unaware of self-injury in youth. Moreover, the diverse range of NSSI behavior during childhood is severely understudied and could contribute to difficulty in determining clinical significance and appropriate treatment for children who engage in self-injury.^50^ Generally, younger children engage in less lethal and less severe self-injury, but this does not indicate lack of suicidal intention or capability.^50^ The earlier-onset group was likely to endorse baseline NSSI with SI, and the later-onset group endorsed more frequent NSSI with greater risk for SA. Thus, results support promoting awareness of NSSI as a serious risk factor for SA in children, particularly when more frequent or more chronically recurring incidents of NSSI or SI are present.

Currently, interventions for childhood NSSI are understudied and lacking. Still, SA risk was present in children with NSSI in this study regardless of which NSSI trajectory children followed. Implications from this study suggest that childhood NSSI is a clinically significant risk factor for SA. Childhood NSSI should warrant clinically indicated suicide risk screening, assessment, and intervention regardless of medical severity. Future research that identifies targets for preventing self-injury through adaptive family interactions and mitigating risk for internalizing symptoms is urgently needed. Until efficacious interventions and preventions are available for children, adaptations of adolescent suicide prevention that incorporate caregivers and reduce self-injury even with high family conflict may be effective. Younger children who engage in self-injury may also benefit from existing child psychopathology interventions, as treating psychological symptoms that commonly co-occur with suicidality can help alleviate suicidality.

To our knowledge, this is the first study to examine SA risk across NSSI trajectories in a high-risk sample of youth (age range 9-14 years) from the United States. Strengths include leveraging a large longitudinal sample of diverse US children and using sophisticated person-centered analyses to identify latent trajectories within the population. Our analysis was limited by ABCD Study measures, which did not include the method, frequency, or severity of NSSI. Dates for NSSI are also not provided, and it is not possible within the ABCD dataset to tease apart temporal proximity of NSSI and SA with the current data. We were unable to include important variables such as neurobiological measures and measures not available at baseline by the ABCD Study, such as emotion regulation and coping. We were also unable to discuss many other important factors that might be associated with NSSI (see Supplement 4, available online, for additional articles). Due to sample size and data use conditions intended to protect against risk of identifying youth participating in the ABCD Study, we needed to collapse some racial and ethnic categories. Predictors of trajectories are also measured at baseline. Lastly, rates of self-injury can be underreported.^45^

Most youth in the present study experienced early NSSI, and 21% had ≥1 SAs before age 16. Results underscore the growing concern that childhood NSSI is a serious risk factor for SA, and certain youth and family characteristics may confer risk for experiencing both NSSI and SA. Implications from this research suggest promoting public awareness of early-onset NSSI as a risk factor for SA, even as young as age 9 years. This research should serve as an important first step in clarifying developmental NSSI trajectories. Continued research examining the risk and protective factors identified in this study are needed to advance more tailored youth suicide prevention efforts.

## CRediT authorship contribution statement

**Amanda J. Thompson:** Writing – review & editing, Writing – original draft, Project administration, Methodology, Investigation, Formal analysis, Data curation, Conceptualization. **Katherine Sarkisian:** Writing – review & editing, Writing – original draft, Conceptualization. **Elyse N. Llamocca:** Writing – review & editing, Writing – original draft, Methodology, Data curation, Conceptualization. **Christopher C. Henrich:** Writing – review & editing, Supervision, Methodology, Formal analysis, Data curation, Conceptualization. **Jennifer L. Hughes:** Writing – review & editing, Supervision, Conceptualization. **Eric A. Youngstrom:** Writing – review & editing, Supervision, Conceptualization. **Donna A. Ruch:** Writing – review & editing, Supervision, Data curation, Conceptualization. **Jeffrey A. Bridge:** Writing – review & editing, Supervision, Conceptualization. **Cynthia A. Fontanella:** Writing – review & editing, Writing – original draft, Supervision, Resources, Methodology, Conceptualization.

## References

[1] Ammerman B.A., Jacobucci R., Kleiman E., Uyeji L., McCloskey M. (2018). The relationship between nonsuicidal self-injury age of onset and severity of self-harm. Suicide Life Threat Behav.

[2] Griep S.K., MacKinnon D.F. (2022). Does nonsuicidal self-injury predict later suicidal attempts? A review of studies. Arch Suicide Res.

[3] Ribeiro J.D., Franklin J.C., Fox K.R. (2016). Self-injurious thoughts and behaviors as risk factors for future suicide ideation, attempts, and death: a meta-analysis of longitudinal studies. Psychol Med.

[4] Burke T.A., Bettis A.H., Walsh R.F.L. (2023). Nonsuicidal self-injury in preadolescents. Pediatrics.

[5] Deville D.C., Whalen D., Breslin F.J. (2020). Prevalence and family-related factors associated with suicidal ideation, suicide attempts, and self-injury in children aged 9 to 10 years. JAMA Netw Open.

[6] Kann L., McManus T., Harris W.A. (2018). Youth Risk Behavior Surveillance—United States, 2017. MMWR Surveill Summ.

[7] Brager-Larsen A., Zeiner P., Klungsøyr O., Mehlum L. (2022). Is age of self-harm onset associated with increased frequency of non-suicidal self-injury and suicide attempts in adolescent outpatients?. BMC Psychiatry.

[8] Cipriano A., Cella S., Cotrufo P. (2017). Nonsuicidal self-injury: a systematic review. Front Psychol.

[9] Zetterqvist M., Jonsson L.S., Landberg Å., Svedin C.G. (2021). A potential increase in adolescent nonsuicidal self-injury during covid-19: a comparison of data from three different time points during 2011–2021. Psychiatry Res.

[10] Swannell S.V., Martin G.E., Page A., Hasking P., St John N.J. (2014). Prevalence of nonsuicidal self-injury in nonclinical samples: systematic review, meta-analysis and meta-regression. Suicide Life Threat Behav.

[11] Barrocas A.L., Giletta M., Hankin B.L., Prinstein M.J., Abela J.R.Z. (2015). Nonsuicidal self-injury in adolescence: longitudinal course, trajectories, and intrapersonal predictors. J Abnorm Child Psychol.

[12] Reinhardt M., Rice K.G., Durán B.S., Kökönyei G. (2022). A person-centered approach to adolescent nonsuicidal self-injury: predictors and correlates in a community sample. J Youth Adolesc.

[13] Wang B., You J., Lin M.P., Xu S., Leung F. (2017). Developmental trajectories of nonsuicidal self-injury in adolescence and intrapersonal/interpersonal risk factors. J Res Adolesc.

[14] Li L., Yang H. (2023). Heterogeneity in adolescents’ non-suicidal self-injury behaviour trajectories based on the group-based trajectory model and a decision tree analysis of family-related determinants. Psychol Res Behav Manag.

[15] Castellví P., Lucas-Romero E., Miranda-Mendizábal A. (2017). Longitudinal association between self-injurious thoughts and behaviors and suicidal behavior in adolescents and young adults: a systematic review with meta-analysis. J Affect Disord.

[16] Whalen D.J., Hennefield L., Elsayed N.M., Tillman R., Barch D.M., Luby J.L. (2022). Trajectories of suicidal thoughts and behaviors from preschool through late adolescence. J Am Acad Child Adolesc Psychiatry.

[17] Somer O., Bildik T., Kabukçu-Başay B., Güngör D., Başay Ö., Farmer R.F. (2015). Prevalence of non-suicidal self-injury and distinct groups of self-injurers in a community sample of adolescents. Social Psychiatry Psychiatr Epidemiol.

[18] Plener P.L., Schumacher T.S., Munz L.M., Groschwitz R.C. (2015). The longitudinal course of non-suicidal self-injury and deliberate self-harm: a systematic review of the literature. Borderline Personal Disord Emot Dysregul.

[19] Liu R.T., Walsh R.F.L., Sheehan A.E., Cheek S.M., Sanzari C.M. (2022). Prevalence and correlates of suicide and nonsuicidal self-injury in children: a systematic review and meta-analysis. JAMA Psychiatry.

[20] Nock M.K., Prinstein M.J. (2004). A functional approach to the assessment of self-mutilative behavior. J Consult Clin Psychol.

[21] Pollak O.H., D’Angelo E.J., Cha C.B. (2020). Does function predict persistence? Nonsuicidal self-injury among adolescents during and after hospitalization. Psychiatry Res.

[22] De Luca L., Pastore M., Palladino B.E., Reime B., Warth P., Menesini E. (2023). The development of non-suicidal self-injury (NSSI) during adolescence: a systematic review and Bayesian meta-analysis. J Affect Disord.

[23] Hamza C.A., Willoughby T., Heffer T. (2015). Impulsivity and nonsuicidal self-injury: a review and meta-analysis. Clin Psychol Rev.

[24] Beauchaine T.P., Hinshaw S.P., Bridge J.A. (2019). Nonsuicidal self-injury and suicidal behaviors in girls: the case for targeted prevention in preadolescence. Clin Psychol Sci.

[25] Luby J.L., Whalen D., Tillman R., Barch D.M. (2019). Clinical and psychosocial characteristics of young children with suicidal ideation, behaviors, and nonsuicidal self-injurious behaviors. J Am Acad Child Adolesc Psychiatry.

[26] Paul E., Ortin A. (2019). Correlates of suicidal ideation and self-harm in early childhood in a cohort at risk for child abuse and neglect. Arch Suicide Res.

[27] Lockwood J., Townsend E., Daley D., Sayal K. (2020). Impulsivity as a predictor of self-harm onset and maintenance in young adolescents: a longitudinal prospective study. J Affect Disord.

[28] Nelson B.W., Pollak O.H., Clayton M.G., Telzer E.H., Prinstein M.J. (2024). An RDoC-based approach to adolescent self-injurious thoughts and behaviors: the interactive role of social affiliation and cardiac arousal. Dev Psychopathol.

[29] Westlund Schreiner M., Klimes-Dougan B., Begnel E.D., Cullen K.R. (2015). Conceptualizing the neurobiology of non-suicidal self-injury from the perspective of the Research Domain Criteria Project. Neurosci Biobehav Rev.

[30] Nock M.K., Joiner T.E., Gordon K.H., Lloyd-Richardson E., Prinstein M.J. (2006). Non-suicidal self-injury among adolescents: diagnostic correlates and relation to suicide attempts. Psychiatry Res.

[31] O’Loughlin C., Burke T.A., Ammerman B.A. (2021). Examining the time to transition from nonsuicidal self-injury to suicide attempt: a brief report. Crisis.

[32] Whitlock J., Muelenkamp J., Eckenrode J. (2013). Nonsuicidal self-injury as a gateway to suicide in young adults. Journal of Adolescent Health.

[33] Kiekens G., Hasking P., Boyes M. (2018). The associations between non-suicidal self-injury and first onset suicidal thoughts and behaviors. Journal of Affective Disorders.

[34] Bryan C.J., Bryan A.O., May A.M., Klonsky E.D. (2015). Trajectories of suicide ideation, nonsuicidal self-injury, and suicide attempts in a nonclinical sample of military personnel and veterans. Suicide Life Threat Behav.

[35] Meza J.I., Owens E.B., Hinshaw S.P. (2021). Childhood predictors and moderators of lifetime risk of self-harm in girls with and without attention-deficit/hyperactivity disorder. Dev Psychopathol.

[36] van de Schoot R., Sijbrandij M., Winter S.D., Depaoli S., Vermunt J.K. (2017). The GRoLTS-Checklist: guidelines for reporting on latent trajectory studies. Struct Equ Modeling.

[37] Barch D.M., Albaugh M.D., Avenevoli S. (2018). Demographic, physical and mental health assessments in the Adolescent Brain and Cognitive Development Study: rationale and description. Dev Cogn Neurosci.

[38] Kaufman J., Birmaher B., Brent D. (1997). Schedule for Affective Disorders and Schizophrenia for School-Age Children–Present and Lifetime version (K-SADS-PL): Initial reliability and validity data. J Am Acad Child Adolesc Psychiatry.

[39] Achenbach T.M. (1991).

[40] DeJoseph M.L., Herzberg M.P., Sifre R.D., Berry D., Thomas K.M. (2022). Measurement matters: An individual differences examination of family socioeconomic factors, latent dimensions of children’s experiences, and resting state functional brain connectivity in the ABCD sample. Dev Cogn Neurosci.

[41] Whiteside S.P., Lynam D.R., Miller J.D., Reynolds S.K. (2005). Validation of the UPPS impulsive behaviour scale: a four-factor model of impulsivity. Eur J Pers.

[42] Carver C.S., White T.L. (1994). Behavioral inhibition, behavioral activation, and affective responses to impending reward and punishment: the BIS/BAS scales. J Pers Soc Psychol.

[43] Barber B.K., Olsen J.E., Shagle S.C. (1994). Associations between parental psychological and behavioral control and youth internalized and externalized behaviors. Child Dev.

[44] Moos R.H. (1974). Family Environment Scale (FES).

[45] Klimes-Dougan B., Mirza S.A., Babkin E., Lanning C. (2022). Biased reporting of past self-injurious thoughts and behaviors: A literature review. J Affect Disord.

[46] Muthén L.K., Muthén B.O. (1998-2010).

[47] Nylund-Gibson K., Grimm R.P., Masyn K.E. (2019). Prediction from latent classes: a demonstration of different approaches to include distal outcomes in mixture models. Struct Equ Modeling.

[48] Rhodes A.E. (2014). Antecedents and sex/gender differences in youth suicidal behavior. World J Psychiatry.

[49] Fong Z.H., Loh W.N.C., Fong Y.J., Neo H.L.M., Chee T.T. (2022). Parenting behaviors, parenting styles, and non-suicidal self-injury in young people: a systematic review. Clin Child Psychol Psychiatry.

[50] Whalen D.J., Luby J.L., Barch D.M. (2018). Highlighting risk of suicide from a developmental perspective. Clin Psychol Sci Pract.

