# Supplementary material for: Developmental Trajectories of Nonsuicidal Self-Injury and Risk for Suicide Attempt
Source: JAACAP Open. 2025 Oct 15;4(1):154–62. doi: 10.1016/j.jaacop.2025.10.004 (PMC12925856; doi:10.1016/j.jaacop.2025.10.004)
Supplement: Supplemental Material [file mmc1.docx]

**Supplemental Materials**

**Supplement 1. Comparison of Children Reporting NSSI vs. No NSSI.**

*Predictors of NSSI Trajectories.* We found a significantly higher proportion of females (52%) compared to males (47%), χ^2^(1, *N*=11,865) = 20.77, *p* =.001, were assigned to the self-harm sample. Results from the one-way ANOVA test found significant differences in the odds of endorsing NSSI across racial groups, F(3, 11859)=8.13, *p*<001. Post-hoc multiple comparisons found White compared to Black youth (*MD* =.05), 95% CI [.03, .07], *p<*.001, Hispanic compared to Black youth (*MD* =.03), 95% CI [.01, .06], *p=*.01, and the combined group of other racial groups compared to Black youth (*MD* = .06) 95% CI [.03, .09], *p*<.001, were more likely to endorse NSSI during the study period.

*Missing data across samples.* We found significant differences in the proportion of youth with missing data on self-harm across the study period. We found that 44% of the sample with NSSI and 36% of the sample without self-harm had complete data on all self-harm assessments, with a higher mean number of missing self-harm data across the study follow-up in the typical sample (*M*=0.93, *SD*=.01) compared to the self-harm sample (*M*=0.75, *SD*=.02), *t*(11875,) = 8.66, *p*<.001.

| **Table S1**  *Mean Comparisons between those without and those with NSSI* | | | | | | | | |  |
| --- | --- | --- | --- | --- | --- | --- | --- | --- | --- |
| Predictor | *t* | df | *p* | MD | SE | Cohen’s d | Cohen’s d 95% CI | |  |
| Internalizing Problems | -19.76 | 11855 | <.001 | -4.64 | .24 | -.44 | [-.49, | -.40] |  |
| Behavioral Activation | -3.23 | 11841 | <.001 | -0.05 | .01 | -.08 | [-.30, | -.21] |  |
| Behavioral Inhibition | -11.39 | 11841 | <.001 | -0.18 | .02 | -.08 | [-.12, | -.03] |  |
| Caregiver Warmth | 12.79 | 11839 | <.001 | 0.08 | .00 | .29 | [.24, | .33] |  |
| Family Conflict | -10.85 | 11836 | <.001 | -0.47 | .04 | -.29 | [-.30, | -.22] |  |
| Ideation | -25.06 | 11834 | <.001 | -0.14 | .01 | -.63 | [-.61, | -.52] |  |
| Material Deprivation | -6.36 | 11841 | <.001 | -0.02 | .00 | -.16 | [-.20, | -.12] |  |
| Negative Urgency | -13.80 | 11839 | <.001 | -0.82 | .06 | -.32 | [-.36, | -.26] |  |

**Supplement 2. Measurement Definition**

Through the 4-year-follow-up, the ABCD study asked children about lifetime history and current self-harm. Children completed the K-SADS-PL at each assessment; caregivers completed the suicidality module at every other assessment. Because research participants often do not disclose lifetime self-injury history at follow-up assessments,^52,53^ we used a combination of any positive endorsement from child and caregiver report to define NSSI and SA on the K-SADS-PL suicidality module. Although there are known discrepancies between parent and child reports of self-harm, because youth may not disclose at follow-up assessments due to recall bias or fear of safety protocols, we used both reporters.

ABCD asked lifetime history questions about NSSI and SA at each assessment but not whether these experiences occurred since the last assessment. Other modules of the KSADS, like the substance use module, do ask whether the experience occurred since the last time the child interacted with the researchers (for more details, see ABCD 5.1 data release notes). Positive endorsements of lifetime history (ever occurred >2 weeks prior to assessment) that were not current (defined as within the past 2 weeks of the assessment) could indicate self-harm occurred between the previous and current assessment. For example, a child may have experienced NSSI since they last participated in an assessment, but the NSSI behavior is no longer current when participating in the follow-up assessment. Similarly, children might endorse lifetime history at baseline and again at a follow-up, and this second lifetime history endorsement at follow-up could indicate new or continuing NSSI or persistent/ chronic duration of NSSI that began earlier. Other children may not endorse lifetime history at follow up despite having endorsed NSSI at prior assessments. This scenario can be applied to SI and SA as well. Thus, lifetime history and current self-harm cannot be disentangled as a separate event in the current data (e.g., more than one SA, or NSSI that resolved then was present again later) for youth positively endorsing both current and lifetime history within a single assessment appointment. Thus, only the first instance of lifetime history and all endorsements of current self-injury for NSSI and SA respectively were collapsed into new variables defined as NSSI and SA that had occurred since the last assessment respectively. Other research examining repeated SA using ABCD data has taken a similar approach.^54^

**Supplement 3. Missing data within the Study Sample.**

Within the self-harm sample, there were no significant differences in total mean number of missing data for self-harm between males and females, *t*(2687) = 0.29, *p*=.73. There were significant differences in the amount of missing data on self-harm between racial groups, F=(3, 2684) 16.93, *p* <.001. Post-hoc mean comparisons found Black children higher mean number of missing data on self-harm compared to White children (*MD*=-.42, p<.001) and Hispanic children (*MD*=-.30, p<.001) and other communities of color (*MD*=-.30, P<.001). Those who entered the study without a baseline lifetime history of NSSI had significantly lower rates of missingness compared to those who did not enter the study with a lifetime history of NSSI, *t*(2687) = -5.05, *p*<.001, MD=-.18.

| **Table S2**  ABCD Variables Used for Defining SA, NSSI, and SI  ABCD File names: mh_y_ksads_ss, mh_p_ksads_ss  The more explicit details about the questions for each item can be found on the ABCD study website: https://abcdstudy.org/scientists/ |
| --- |

| **Parent Variable** | **Child Variable** | **Label** | **Definition** |
| --- | --- | --- | --- |
| ksads_23_956_p | ksads_23_956_t | Diagnosis - SelfInjuriousBehaviorwithoutsuicidalintentPast | NSSI |
| ksads2_23_916_p | ksads2_23_916_t | Diagnosis - SelfInjuriousBehaviorwithoutsuicidalintentPast | NSSI |
| ksads_23_945_p | ksads_23_945_t | Diagnosis - SelfInjuriousBehaviorwithoutsuicidalintentPresent | NSSI |
| ksads2_23_905_p | ksads2_23_905_t | Diagnosis - SelfInjuriousBehaviorwithoutsuicidalintentPresent | NSSI |
| ksads_23_964_p | ksads_23_964_t | Diagnosis - AbortedAttemptPast | SA |
| ksads2_23_924_p | ksads2_23_924_t | Diagnosis - AbortedAttemptPast | SA |
| ksads_23_963_p | ksads_23_963_t | Diagnosis - InterruptedAttemptPast | SA |
| ksads2_23_923_p | ksads2_23_923_t | Diagnosis - InterruptedAttemptPast | SA |
| ksads_23_965_p | ksads_23_965_t | Diagnosis - SuicideAttemptPast | SA |
| ksads2_23_925_p | ksads2_23_925_t | Diagnosis - SuicideAttemptPast | SA |
| ksads_23_822_p | ksads_23_822_t | Symptom - Aborted or interrupted suicide attempts Past | SA |
| ksads2_23_771_p | ksads2_23_771_t | Symptom - Aborted or interrupted suicide attempts, Past | SA |
| ksads_23_825_p | ksads_23_825_t | Symptom - Expect could die from suicide attempt past | SA |
| ksads2_23_774_p | ksads2_23_774_t | Symptom - Expect could die from suicide attempt, past | SA |
| ksads_23_823_p | ksads_23_823_t | Symptom - Number of suicide attempts Past | SA |
| ksads2_23_772_p | ksads2_23_772_t | Symptom - Number of suicide attempts, Past | SA |
| ksads_23_816_p | ksads_23_816_t | Symptom - Self-injury intent to die Past | SA |
| ksads2_23_765_p | ksads2_23_765_t | Symptom - Self-injury, intent to die, Past | SA |
| ksads_23_817_p | ksads_23_817_t | Symptom - Self-Injury thought could die from behavior Past | SA |
| ksads2_23_766_p | ksads2_23_766_t | Symptom - Self-Injury, thought could die from behavior, Past | SA |
| ksads_23_150_p | ksads_23_150_t | Symptom - Suicidal Attempt Past | SA |
| ksads2_23_141_p | ksads2_23_141_t | Symptom - Suicidal Attempt, Past | SA |
| ksads_23_824_p | ksads_23_824_t | Symptom - Suicide attempt method Past | SA |
| ksads2_23_773_p | ksads2_23_773_t | Symptom - Suicide attempt, method, Past | SA |
| ksads_23_953_p | ksads_23_953_t | Diagnosis - AbortedAttemptPresent | SA |
| ksads2_23_913_p | ksads2_23_913_t | Diagnosis - AbortedAttemptPresent | SA |
| ksads_23_952_p | ksads_23_952_t | Diagnosis - InterruptedAttemptPresent | SA |
| ksads2_23_912_p | ksads2_23_912_t | Diagnosis - InterruptedAttemptPresent | SA |
| ksads_23_954_p | ksads_23_954_t | Diagnosis - SuicideAttemptPresent | SA |
| ksads2_23_914_p | ksads2_23_914_t | Diagnosis - SuicideAttemptPresent | SA |
| ksads_23_813_p | ksads_23_813_t | Symptom - Aborted or interrupted suicide attempts Present | SA |
| ksads2_23_762_p | ksads2_23_762_t | Symptom - Aborted or interrupted suicide attempts, Present | SA |
| ksads_23_814_p | ksads_23_814_t | Symptom - Method of actual suicide attempt Present | SA |
| ksads2_23_763_p | ksads2_23_763_t | Symptom - Method of actual suicide attempt, Present | SA |
| ksads_23_807_p | ksads_23_807_t | Symptom - Self-injury intent to die Present | SA |
| ksads2_23_756_p | ksads2_23_756_t | Symptom - Self-injury, intent to die, Present | SA |
| ksads_23_808_p | ksads_23_808_t | Symptom - Self-Injury thought could die from behavior Present | SA |
| ksads2_23_757_p | ksads2_23_757_t | Symptom - Self-Injury, thought could die from behavior, Present | SA |
| ksads_23_149_p | ksads_23_149_t | Symptom - Suicidal Attempt Present | SA |
| ksads2_23_140_p | ksads2_23_140_t | Symptom - Suicidal Attempt, Present | SA |
| ksads_23_815_p | ksads_23_815_t | Symptom - Suicide attempt thought could die Present | SA |
| ksads2_23_764_p | ksads2_23_764_t | Symptom - Suicide attempt, thought could die, Present | SA |
| ksads_23_962_p | ksads_23_962_t | Diagnosis - PreparatoryActionstowardimminentSuicidalbehaviorPast | SI |
| ksads2_23_922_p | ksads2_23_922_t | Diagnosis - PreparatoryActionstowardimminentSuicidalbehaviorPast | SI |
| ksads_23_960_p | ksads_23_960_t | Diagnosis - SuicidalideationActiveintentPast | SI |
| ksads2_23_920_p | ksads2_23_920_t | Diagnosis - SuicidalideationActiveintentPast | SI |
| ksads_23_959_p | ksads_23_959_t | Diagnosis - SuicidalideationActivemethodPast | SI |
| ksads2_23_919_p | ksads2_23_919_t | Diagnosis - SuicidalideationActivemethodPast | SI |
| ksads_23_958_p | ksads_23_958_t | Diagnosis - SuicidalideationActivenonspecificPast | SI |
| ksads2_23_918_p | ksads2_23_918_t | Diagnosis - SuicidalideationActivenonspecificPast | SI |
| ksads_23_961_p | ksads_23_961_t | Diagnosis - SuicidalideationActiveplanPast | SI |
| ksads2_23_921_p | ksads2_23_921_t | Diagnosis - SuicidalideationActiveplanPast | SI |
| ksads_23_957_p | ksads_23_957_t | Diagnosis - SuicidalideationPassivePast | SI |
| ksads2_23_917_p | ksads2_23_917_t | Diagnosis - SuicidalideationPassivePast | SI |
| ksads2_23_99_p | ksads2_23_99_t | SuicidalideationActivemethodPast | SI |
| ksads_23_821_p | ksads_23_821_t | Symptom - Suicidal behavior made preparations Past | SI |
| ksads2_23_770_p | ksads2_23_770_t | Symptom - Suicidal behavior, made preparations, Past | SI |
| ksads_23_818_p | ksads_23_818_t | Symptom - Suicidal ideation thought of method Past | SI |
| ksads2_23_767_p | ksads2_23_767_t | Symptom - Suicidal ideation thought of method, Past | SI |
| ksads_23_819_p | ksads_23_819_t | Symptom - Suicidal ideation intent to act Past | SI |
| ksads2_23_768_p | ksads2_23_768_t | Symptom - Suicidal ideation, intent to act, Past | SI |
| ksads_23_148_p | ksads_23_148_t | Symptom - Suicidal Ideation Past | SI |
| ksads2_23_139_p | ksads2_23_139_t | Symptom - Suicidal Ideation, Past | SI |
| ksads_23_820_p | ksads_23_820_t | Symptom - Suicidal ideation specific plan Past | SI |
| ksads2_23_769_p | ksads2_23_769_t | Symptom - Suicidal ideation, specific plan, Past | SI |
| ksads_23_146_p | ksads_23_146_t | Symptom - Wishes/Better off dead Past | SI |
| ksads2_23_137_p | ksads2_23_137_t | Symptom - Wishes/Better off dead, Past | SI |
| ksads_23_951_p | ksads_23_951_t | Diagnosis - PreparatoryActionstowardimminentSuicidalbehaviorPresent | SI |
| ksads2_23_911_p | ksads2_23_911_t | Diagnosis - PreparatoryActionstowardimminentSuicidalbehaviorPresent | SI |
| ksads_23_949_p | ksads_23_949_t | Diagnosis - SuicidalideationActiveintentPresent | SI |
| ksads2_23_909_p | ksads2_23_909_t | Diagnosis - SuicidalideationActiveintentPresent | SI |
| ksads_23_948_p | ksads_23_948_t | Diagnosis - SuicidalideationActivemethodPresent | SI |
| ksads2_23_908_p | ksads2_23_908_t | Diagnosis - SuicidalideationActivemethodPresent | SI |
| ksads_23_947_p | ksads_23_947_t | Diagnosis - SuicidalideationActivenonspecificPresent | SI |
| ksads2_23_907_p | ksads2_23_907_t | Diagnosis - SuicidalideationActivenonspecificPresent | SI |
| ksads_23_950_p | ksads_23_950_t | Diagnosis - SuicidalideationActiveplanPresent | SI |
| ksads2_23_910_p | ksads2_23_910_t | Diagnosis - SuicidalideationActiveplanPresent | SI |
| ksads_23_946_p | ksads_23_946_t | Diagnosis - SuicidalideationPassivePresent | SI |
| ksads2_23_906_p | ksads2_23_906_t | Diagnosis - SuicidalideationPassivePresent | SI |
| ksads_23_812_p | ksads_23_812_t | Symptom - Suicidal behavior made preparations Present | SI |
| ksads2_23_761_p | ksads2_23_761_t | Symptom - Suicidal behavior, made preparations, Present | SI |
| ksads_23_809_p | ksads_23_809_t | Symptom - Suicidal ideation thought of method Present | SI |
| ksads2_23_758_p | ksads2_23_758_t | Symptom - Suicidal ideation thought of method, Present | SI |
| ksads_23_810_p | ksads_23_810_t | Symptom - Suicidal ideation intent to act Present | SI |
| ksads2_23_759_p | ksads2_23_759_t | Symptom - Suicidal ideation, intent to act, Present | SI |
| ksads_23_147_p | ksads_23_147_t | Symptom - Suicidal Ideation Present | SI |
| ksads2_23_138_p | ksads2_23_138_t | Symptom - Suicidal Ideation, Present | SI |
| ksads_23_811_p | ksads_23_811_t | Symptom - Suicidal ideation specific plan Present | SI |
| ksads2_23_760_p | ksads2_23_760_t | Symptom - Suicidal ideation, specific plan, Present | SI |
| ksads_23_145_p | ksads_23_145_t | Symptom - Wishes/Better off dead Present | SI |
| ksads2_23_136_p | ksads2_23_136_t | Symptom - Wishes/Better off dead, Present | SI |

| **Table S3. Unconditional Models** | |  |  |  |  |  |  |  | |  | | | |
| --- | --- | --- | --- | --- | --- | --- | --- | --- | --- | --- | --- | --- | --- |
| *Unconditional Latent Growth Curve Models* | | | | | | | | | | |  |  |  |
|  | AIC | | BIC | CAIC | intercept | slope | quadratic |  | |  | | |  |
| Linear | 10976.78 | | 11005.95 | 10990.06 | 1.13** | 0.30** | _ |  | |  | | |  |
| Quadratic | 10914.56 | | 10967.07 | 10938.47 | 3.27 ** | 4.52 ** | 0.26** |  | |  | | |  |
| **p*<.05, ***p*<.01 | | | | | | | | |  | | |  |  |

**Supplement 4. Additional Recommended Readings**

The topic of NSSI and SA is an important and growing research area with important citations that we were unable to discuss in the manuscript, but we felt important to recommend as additional readings. These citations can be found below.

1. Adrian M, Ziegler M, Kessler H, et al. Genetic predictors of suicide attempts and nonsuicidal self-injury: A systematic review. J Affect Disord. 2019; 246:1-10. doi:10.1016/j.jad.2018.12.071.
2. Kaess M, Parzer P, Mattern M, et al. Genetic and environmental influences on nonsuicidal self-injury and suicide attempts in adolescents. J Am Acad Child Adolesc Psychiatry. 2021;60(5):567-576. doi:10.1016/j.jaac.2021.01.020.
3. Mason W, Birmaher B, Goldstein B, et al. Longitudinal predictors of suicide attempts in adolescents with major depressive disorder. J Am Acad Child Adolesc Psychiatry. 2025;64(1):45-53. doi:10.1016/j.jaac.2024.08.013.
4. Wang Y, Lai J, Hu C, et al. Non-suicidal self-harm is linked to suicidal thoughts in Chinese adolescents with mood disorders: A cross-sectional report. J Zhejiang Univ Sci B. 2021;22(3):233-240. doi:10.1631/jzus.B2000679.
5. Giletta M, Scholte RHJ, Engels RCME, et al. The interplay between peer victimization and nonsuicidal self-injury in adolescence. J Abnorm Child Psychol. 2015;43(5):885-894. doi:10.1007/s10802-015-0019-3.
6. Ortin-Peralta C, Sheftall AH, Osborn A, et al. Severity and transition of suicidal behaviors in childhood: Sex, racial, and ethnic differences in the Adolescent Brain Cognitive Development (ABCD) Study. J Adolesc Health. 2023;73(4):724-730. doi:10.1016/j.jadohealth.2023.05.026.
7. Ortin-Peralta A, Schiffman A, Malik J, Polanco-Roman L, Hennefield L, Luking K. Negative and positive urgency as pathways in the intergenerational transmission of suicide risk in childhood. Front Psychiatry. 2024 Sep 23;15:1417991. doi: 10.3389/fpsyt.2024.1417991. PMID: 39376969; PMCID: PMC11456838.
8. Zhang Y, Gong L, Feng Q, et al. Association between negative life events through mental health and non-suicidal self-injury with young adults: Evidence for sex-moderated correlation. BMC Psychiatry. 2024;24:466. doi:10.1186/s12888-024-05880-33.
